# Ultrasonic liquid-phase catalysis for enhanced power generation in Al-based bioelectrolyte batteries

**DOI:** 10.1016/j.ultsonch.2026.107822

**Published:** 2026-03-15

**Authors:** Huiyu Huang, Jia Yin, Shuo Zhang, Quanquan Yang, Zhong Chen, Qiang Tang, Xiaomin Qi, Songfei Su, Jinyan Chen, Hao Chen, Kan Zhu, Shengling Qu, Pengzhan Liu

**Affiliations:** a Jiangsu Key Laboratory of Advanced Manufacturing Technology, Faculty of Mechanical and Material Engineering, Huaiyin Institute of Technology, Huaian 223003, China; b State Key Laboratory of Mechanics and Control for Aerospace Structures, Nanjing University of Aeronautics and Astronautics, Nanjing 210016, China; c School of Mechanical and Automotive Engineering, Anhui Polytechnic University, Wuhu 241000, China; d School of Mechanical Engineering, Nanjing Institute of Technology, Nanjing 211167, China; e School of Automotive Engineering, Changzhou Institute of Technology, Changzhou 213032, China; f School of Mechanical and Aerospace Engineering, Nanyang Technological University, 639798, Singapore; g School of Materials Science and Intelligent Engineering, Nanjing University, Suzhou 215163, China

**Keywords:** Al-based battery, Bioelectrolyte, Ultrasound, Catalysis, Acoustics

## Abstract

Al-based batteries have been particularly attractive for integration with bioelectrolytes such as sweat and tears, enabling portable and biocompatible power sources. However, their practical applications are severely constrained by sluggish mass transfer and reaction kinetics at the catalytic electrode interface. In this study, we present a compact ultrasound-catalyzed Al-based bioelectrolyte battery (ABBB) to overcome this limitation, for which the catalysis mechanisms arise from the synergistic effects of acoustic pressure, acoustic streaming, and ultrasonic stirring for jointly accelerating interfacial mass transport and electrode reaction kinetics. Experimental results show that the ultrasonic liquid-phase catalysis can dramatically improve battery discharge performance, with peak power enhanced by higher than one order of amplitude (10-fold). Notably, when the artificial sweat mimicking human sweat composition is employed as the electrolyte, the peak power can be enhanced by approximately 5-fold under the ultrasonic excitation, highlighting its feasibility for practical biofluid-powered systems. This work establishes a novel physical-field catalytic strategy for boosting ABBB performance and provides a solid foundation for their applications in self-powered biosensors, wearable medical devices, and green electronic technologies.

## 1 Introduction

As global energy demand increasingly shifts toward low-carbon and sustainable solutions, aluminium (Al)-based batteries (ABBs) have emerged as promising candidates for next-generation energy storage systems, owing to the abundance of Al resources, high theoretical energy density, recyclability, low cost, manufacturability, and overall sustainability [1], [2]. However, the widespread deployment of ABBs has severely been constrained by their dependence on conventional electrolytes [3], [4], such as expensive and flammable ionic liquids or toxic organic solvents, which limits their suitability for emerging applications in wearable and bio-integrated electronics [5], [6]. Recently, the exploration of biocompatible fluids such as sweat, urine, and tears to be used as electrolytes for powering bioelectronic devices has opened a new and attractive avenue [7], [8], [9]. These bioelectrolytes are not only readily accessible and inherently safe but also contain a rich variety of organic molecules including glucose, lactic acid, ascorbic acid, urea, uric acid, as well as inorganic ions such as NaCl and KCl. Such components can effectively regulate discharge behaviors of ABBs by suppressing Al electrode corrosion, thereby offering an unique opportunity for the development of environmentally-friendly and wearable micro-power sources [10], [11], [12].

The discharge performance of ABBs hinges on the electrochemical reaction kinetics at the electrodes, particularly the catalytic activities toward the oxygen reduction reactions (ORRs) at the cathodes, which can be achieved through the development of highly efficient catalyst materials or electrode structures with large specific surface areas [13], [14], [15], [16]. Accordingly, high-performance catalysts typically include noble metals, carbon-based materials, transition metal oxides, and single-atom catalysts [17], [18], [19], [20], while electrode architectures with high specific surface areas generally extend the three-phase reaction interface (TPRI) from a two-dimensional (2D) plane to a three-dimensional (3D) region, thereby enhancing catalytic activity [21], [22], [23]. These advanced catalytic materials and electrode structures effectively enhance the discharge performance of ABBs by increasing the density of active sites at the cathode interface and improving electronic conductivity [24], [25]. However, these strategies still face significant scientific and technological challenges in the development of ABBs, including high cost, complex fabrication processes, limited stability, and short operational lifetimes, which hinder their large-scale applications and further development. Moreover, in bioelectrolyte systems such as sweat-, tear-, and urine-based electrolytes, the inherently slow mass transport of biomolecules at the electrode–electrolyte interface, together with the accumulation of reaction byproducts on the electrode surface, severely suppresses the ORR kinetics at the TPRI and compromises the long-term stability of the cathodic catalytic performance [26], [27], [28]. Consequently, there is an urgent need to develop effective physical catalytic strategies capable of enhancing interfacial mass transport and dynamically regulating the micro-environment at the electrode interface.

Ultrasound technology, as an effective physical field-assisted approach, has widely been employed to enhance the discharge performance of various battery systems [29], [30], [31], [32], [33], [34], [35], [36], [37], [38]. Representative studies include improving the discharge performance of Al-air batteries by inducing electrolyte circulation through ultrasonic capillary effects [29], enhancing the performance of Al/Mg-air batteries by airborne ultrasonic catalysis [30], promoting battery discharge behavior by fabricating porous catalytic electrode structures using the ultrasound-assisted material processing [38], and boosting the performance of lithium (Li) metal batteries through suppressing Li dendrite growth using the acoustic streaming generated by surface acoustic waves (SAWs) [39]. However, the existing ultrasound-assisted strategies have predominantly focused on electrolyte flow enhancement, airborne ultrasonic catalysis, or material preparation processes. In most cases, the ultrasonic devices are not integrated with the battery into a unified system, which significantly limits system miniaturization and practical applicability. By contrast, an integrated ultrasound-assisted battery system enables *in-situ* physical catalysis by directly generating an ultrasonic field at the catalytic electrode within the bioelectrolyte.

In this work, we propose a high-performance Al-based bioelectrolyte battery (ABBB) enabled by an ultrasonic liquid-phase catalysis strategy, in which an ultrasonic excitation is externally applied to the battery system. We systematically elucidate how ultrasonic physical effects catalyze cathodic processes, thereby enhancing discharge performance through the improved interfacial mass transport and the dynamic modulation of electrode micro-environment. This approach effectively addresses the intrinsic limitations of bioelectrolyte-based ABBs and expands the application of physical field-assisted catalysis in electrochemical energy systems, offering a promising pathway toward sustainable and biocompatible power sources for next-generation wearable and implantable electronics.

## 2 Methods and experiments

### 2.1 Principle, device, and measurement of the ultrasound-catalyzed ABBB

**Fig. 1:**
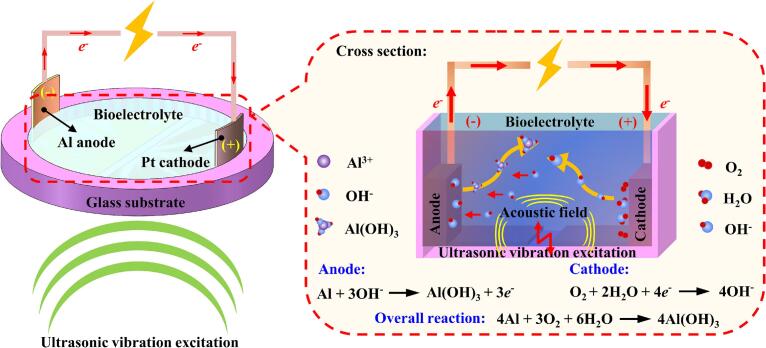
Principle schematic of the ABBB catalyzed by ultrasound.

Fig. 1 schematically illustrates the working principle of the ultrasound-catalyzed ABBB. In the experiments, different bioelectrolytes provide distinct electrochemical reaction environments. During battery operation, the Platinum (Pt) electrode serves as the catalytic cathode; oxygen, supplied from ambient air and dissolved in the bioelectrolyte, undergoes a reduction reaction at the cathode by accepting electrons from the external circuit to generate hydroxide ions (OH^–^). The generated OH^–^ ions subsequently diffuse through the bioelectrolyte toward the Al anode, where they react with Al to form Al hydroxide (Al(OH)_3_) while releasing electrons. These electrons are transported back to the cathode through the external circuit, sustaining the continuous electrochemical reaction process. The corresponding electrochemical reactions of ABBB are summarized as follows [30]:

(1) $$\text{Cathode}:O_{2}+2H_{2}O+4e^{-}\to 4OH^{-}$$

(2) $$\text{Anode}:Al+3OH^{-}\to Al{\left(\mathrm{OH}\right)}_{3}+3e^{-}$$

(3) $$\text{Overall}:4Al+3O_{2}+6H_{2}O\to 4Al{\left(\mathrm{OH}\right)}_{3}$$

Through incorporating ultrasound, the mechanism of ultrasonic liquid-phase catalysis can be elucidated as follows:

(I) The acoustic pressure field generated at the cathode-electrolyte interface can promote the ORR kinetics, thereby enhancing the intrinsic catalytic activity of cathode electrode [29].

(II) The acoustic streaming induced by ultrasonic excitation plays a dual role in this liquid-phase catalysis. First, the ultrasonic microjets generate localized high-energy flow regions near the cathode surface, which can effectively thin the Nernst diffusion layer and substantially enhance the mass transport of electroactive species from the bulk electrolyte to the electrode surface across the electric double layer [43]. Second, the acoustic streaming can accelerate the diffusion of OH^–^, thereby further increasing the overall electrochemical reaction rate [44], [45].

(III) Moreover, the ultrasonic stirring effect can effectively suppress the accumulation and deposition of Al hydroxide flocculates at the cathode interface, preventing electrode passivation and thus improving the operational stability and service lifetime of battery [26].

Benefiting from the synergistic effects of acoustic pressure, acoustic streaming, and ultrasonic stirring, the catalytic performance of cathode electrode can be significantly enhanced. Consequently, this ultrasonic liquid-phase catalysis leads to a pronounced improvement in the discharge performance of battery.

**Fig. 2:**
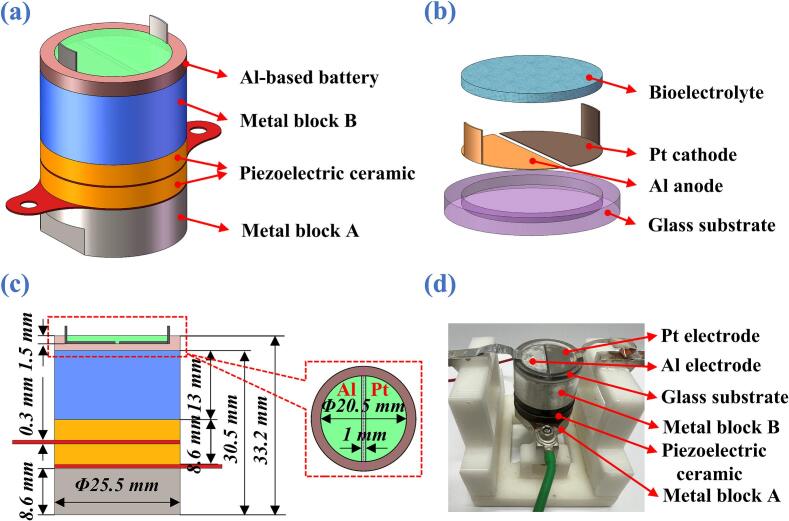
(a) Structure diagram to show the integration between the ultrasonic transducer and the battery unit. (b) Structure diagram of the ABBB. (c) Dimensions of the ultrasound-assisted ABBB. (d) Prototype photo.

To adapt this ultrasonic liquid-phase catalysis principle into a practical ABBB system, the structure and configuration of an ultrasound-assisted ABBB are illustrated in Fig. 2(a)-Fig. 2(d). The device mainly consists of an ultrasonic transducer (HNC-4AS-2565, Hainertec Co., Ltd.) and an ABBB unit bonded directly onto the surface of the transducer. The ultrasonic transducer has a resonance frequency of 65.1 kHz and dimensions (diameter × height) of *ϕ*25 × 31 mm, with detailed performance parameters summarized in Table S1 (Supplementary Materials). As shown in Fig. 2(b), the ABBB unit comprises a glass substrate (Nantong Shredder Laboratory Equipment Co., Ltd.) with dimensions of *ϕ*25 × 2.7 mm, an Al electrode serving as the anode, a Pt electrode serving as the cathode, and a bioelectrolyte. To accommodate the electrodes and form an electrochemical reaction chamber, a cylindrical cavity with dimensions of *ϕ*20.5 × 1.5 mm is machined into the upper surface of the substrate, resulting in a bioelectrolyte volume of approximately 0.5 mL. The Al and Pt electrodes are semicircular in shape, each with a diameter of 20.5 mm and a thickness of 0.06 mm, and are separated by a gap of 1 mm, as illustrated in Fig. 2(c). A photo of the assembled ultrasound-assisted ABBB prototype is shown in Fig. 2(d), demonstrating the integrated configuration of the ultrasonic transducer and the battery unit.

**Fig. 3:**
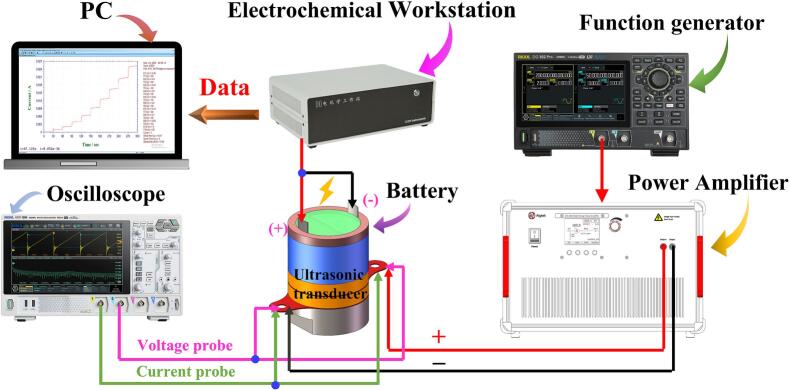
Experimental setup for performance measurement of the ultrasound-catalyzed ABBB.

Fig. 3 illustrates the overall experimental setup used for performance testing of the ultrasound-catalyzed ABBB. A function generator (DG922pro, RIGOL Technologies Co., Ltd.) is employed to control a power amplifier (ATA4052, Xi’an Aigtek Electronic Technology Co., Ltd.), which drives the ultrasonic transducer. Real-time operating states of the ultrasound-catalyzed ABBB, including voltage and current signals, are monitored using an oscilloscope (DHO1074, RIGOL Technologies Co., Ltd.). Meanwhile, the polarization characteristics of battery are measured and recorded using an electrochemical workstation (CHI 760E, Shanghai Chenhua Instrument Co., Ltd.). As such, these instruments constitute an integrated testing platform that enables synchronized ultrasonic excitation, electrochemical characterization, and electrical signal acquisition.

### 2.2 Vibration characterization

**Fig. 4:**
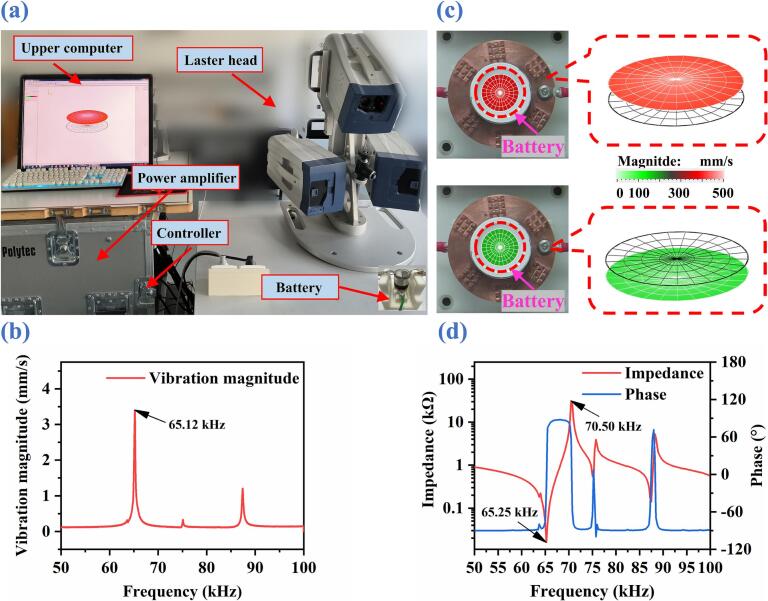
(a) Vibration measurement system using an LDV. (b) Frequency spectrum. (b) Instantaneous vibration displacement distributions on the glass substrate surface at 65.12 kHz and 35.2 V_pp_. (d) Impendence spectrum.

To determine the vibration mode and resonance frequency of the ultrasound-catalyzed ABBB, a laser Doppler vibrometer (LDV) system (PSV-300F-B, Polytec) is employed to characterize the vibration response of the battery glass substrate. Through the experimental setup shown in Fig. 4(a), the frequency-dependent vibration amplitude of the glass substrate surface is shown in Fig. 4(b), from which a pronounced resonance peak is observed at 65.12 kHz. Accordingly, Fig. 4(c) presents the instantaneous vibration displacement distributions on the glass substrate surface at 65.12 kHz and 35.2 V_pp_. A piston-like vibration mode is clearly observed, where the red-colored region corresponds to upward motion toward the laser head, while the green-colored region represents downward motion away from the laser head. To further verify the accuracy of the LDV test, an impedance analysis is performed using an impedance analyzer (4294A, Agilent). As shown in Fig. 4(d), the resonance frequency from the impedance analysis is determined to be 65.25 kHz, which is close to the LDV test result (65.12 kHz).

### 2.3 Chemicals and electrochemical measurements

All chemical materials are of analytical grades and directly prepared into solutions without further purification. The bioelectrolyte is prepared with deionized water (CJL-20–15, Ultrapure Water Co., Ltd.). Sodium chloride (NaCl, ≥99.8%), D(+)-Glucose anhydrous (Glucose), DL-Lactic acid (Lactate, ≥85%), L-Ascorbic acid (AA), and potassium chloride (KCl, ≥99.8%) are purchased from Sinopharm Chemical Reagent Co., Ltd. Urea (≥99.9%) is purchased from Aladdin Reagent Co., Ltd. Uric acid (UA, ≥99.8%) is purchased from Wokai Pharmaceutical Co., Ltd. Artificial sweat is purchased from Deli Chemical Co., Ltd.

In the characterization experiments, NaCl-, glucose-, and lactate-based bioelectrolytes are prepared with concentrations of 1 M, 2 M, and 3 M, respectively. Since glucose solutions reach saturation at approximately 4 M and cannot be further prepared, 3 M is selected as the maximum bioelectrolyte concentration for principle verification in this work. The concentration of bioelectrolyte is calculated using the following relationship:

(4) $$c_{x}=\frac{n_{x}}{V_{x}}=\frac{m_{x}}{M_{x}}\cdot \frac{1}{V_{x}}$$

where *n_x_* is the amount of bioelectrolyte substance, *V_x_* is the bioelectrolyte volume, *m_x_* is the bioelectrolyte mass, and *M_x_* is the molar mass. Furthermore, the concentrations of various components in human sweat span a broad range, including Na^+^ (10–100 mM), Cl^+^ (10–100 mM), K^+^ (1–18.5 mM), glucose (10–200 μM), lactate (5–20 mM), uric acid (2–10 mM), and ascorbic acid (10–50 mM) [40], [41], [42]. Accordingly, for the application-oriented experiments that will be described in Section 3.6, the concentrations of urea, glucose, AA, UA, lactate, KCl, and NaCl are prepared as 30 mM, 50 μM, 20 μM, 4 mM, 5 mM, 10 mM, and 100 mM, respectively.

The electrochemical performance of ABBB is evaluated through polarization measurements, including the current–voltage (*I*-*V*) and current-power (*I*-*P*) curves, as well as the electrochemical impedance spectroscopy (EIS). All electrochemical tests are conducted at room temperature (25 ± 2 °C) using the electrochemical workstation.

The open-circuit voltage (OCV) of ABBB is first recorded to be approximately 0.55 V. Polarization curves are subsequently measured using a potentiostatic step method. Prior to data acquisition, the battery is activated by multiple cyclic voltammetry scans until the peak discharge power becomes stable, in order to remove the native Al oxide film on the anode surface [29]. During polarization testing, the applied voltage is decreased stepwise from 0.55 V to 0 V with a step size of 0.05 V. Each voltage step is maintained for 30 s, and the average current value obtained during the latter half of each step (15–30 s), where a stable current response is achieved, is used to determine the corresponding discharge current. Based on the stable current values at each voltage, the polarization (*I*-*V*) curve is constructed, while the current-power (*I*-*P*) curve is derived from the relationship P=V×I. In addition, EIS is performed at the OCV of 0.55 V over a frequency range from 100 kHz to 0.1 Hz, with an AC perturbation amplitude of 5 mV, to characterize impedance behaviors of ABBB.

### 2.4 Numerical simulations

**Fig. 5:**
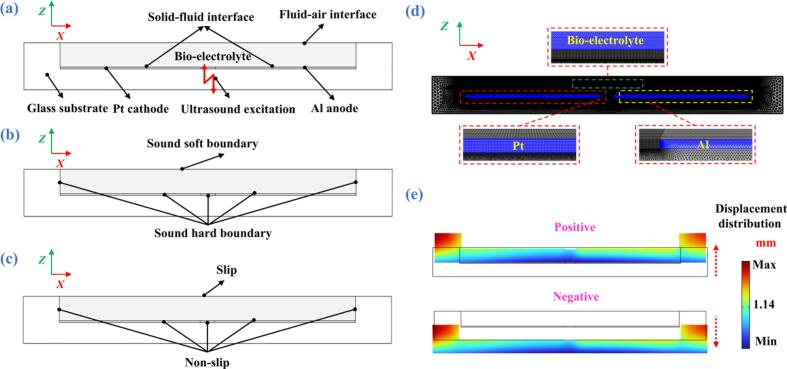
(a) 2D FEM model for numerical simulations of the ABBB unit under ultrasonic excitation. (b) Boundary conditions for simulating the acoustic field. (c) Boundary conditions for simulating the acoustic streaming field. (d) Meshed 2D FEM model. (e) Simulated vibration displacement distributions of the glass substrate.

To elucidate the physical mechanisms by which the ultrasonic liquid-phase catalysis enhances the discharge performance of ABBB, numerical simulations of acoustic and acoustic streaming fields are performed using the finite element method (FEM) software COMSOL Multiphysics 6.2 [43], [44], [45]. In the FEM analysis, a 2D model with the same geometry and dimensions as the experimental battery unit is constructed, as illustrated in Fig. 5(a). The boundary conditions used to simulate the acoustic and acoustic streaming fields are shown in Fig. 5(b) and Fig. 5(c), respectively, and a meshed FEM model is shown in Fig. 5(d).

Using the simulation scheme that is well described in our previous publications as well as the material parameters listed in Table S2, a numerical computation is first performed to analyze the vibration response of the system [43], [44], [45]. Fig. 5(e) shows the computed vibration displacement distribution of the glass substrate. The results indicate that the glass substrate undergoes a piston-like up-and-down vibration mode, which is in excellent agreement with the LDV measurement results (Fig. 4(b)).

## 3 Results and discussion

### 3.1 Performance of the ultrasound-catalyzed ABBB

**Fig. 6:**
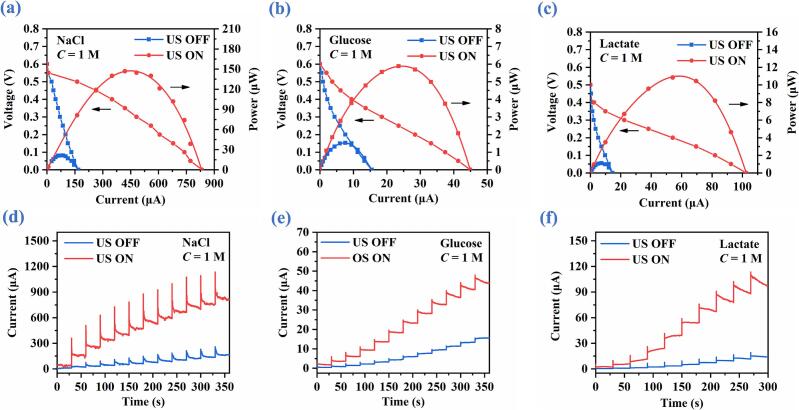
Discharge performance of ABBB with and without ultrasonic liquid-phase catalysis in NaCl, glucose, and lactate bioelectrolytes, respectively. (a), (b) & (c) Polarization and power curves. (d), (e) & (c) Potentiostatic measurement curves.

The *I*-*V*, *I*-*P*, and potentiostatic discharge characteristics of the ultrasound-catalyzed ABBB are evaluated using the 1-M NaCl, glucose, and lactate bioelectrolytes at 65.12 kHz and 35.2 V_pp_, under which the vibration velocity amplitude of the glass substrate is 459.3 mm/s. As presented in Fig. 6, compared with the without-ultrasound (US OFF) condition, both the peak output power and short-circuit current (SCC) are markedly enhanced by ultrasonic excitation. As shown in Fig. 6(a)-Fig. 6(c), for NaCl, glucose, and lactate bioelectrolytes, the peak powers of ABBB are increased by ultrasound from 21.383 mW, 1.522 mW, and 1.101 mW to 146.754 mW, 5.879 mW, and 10.838 mW, corresponding to enhancing discharge performance by 6.86-, 3.86-, and 9.84-fold, respectively. Meanwhile, the SCCs are significantly increased from 169.942 μA, 15.486 μA, and 14.567 μA to 833.747 μA, 44.865 μA, and 102.463 μA, representing increases of 4.9-, 2.9-, and 7.0-fold, respectively.

Furthermore, the potentiostatic discharge curves shown in Fig. 6(d)-Fig. 6(f) demonstrate that the ultrasonic catalysis consistently promotes the discharge current at different applied voltages, with the corresponding voltage step protocol described in Section 2.3. Overall, these results indicate that the ultrasonic liquid-phase catalysis provides a general and effective approach to increasing the operating current and output power of ABBB.

### 3.2 Principle analysis

#### 3.2.1 FEM simulations

**Fig. 7:**
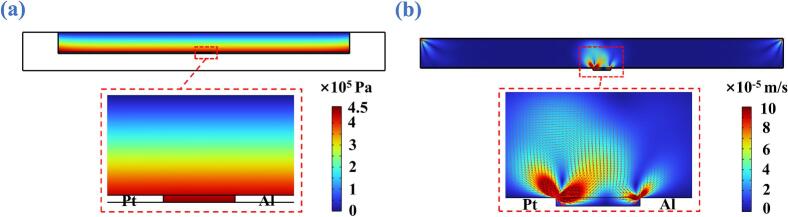
(a) Simulated distribution of acoustic pressure amplitude in the bioelectrolyte domain. (d) Simulated acoustic streaming field in the bioelectrolyte domain.

Under 65.12 kHz and 459.3 mm/s, Fig. 7(a) shows the simulated distribution of acoustic pressure amplitude in the bioelectrolyte domain. It is observed that a strong pressure field is generated in the region between the Al and Pt electrodes, particularly within the inter-electrode gap. Such an intensified acoustic pressure field can effectively promote electrochemical reaction kinetics at the catalytic electrode interface, thereby enhancing the intrinsic catalytic activity of cathode electrode [29]. Subsequently, Fig. 7(b) shows the simulated acoustic streaming field in the bioelectrolyte domain. It is observed that acoustic streaming eddies are generated at the interface of each Al/Pt electrode, which plays a dual role in this ultrasonic liquid-phase catalysis method. One is that the high-energy streaming regions near the cathode surface can effectively thin the Nernst diffusion layer and substantially enhance the mass transport of electroactive species [43]. The other one is that the acoustic streaming can also accelerate the diffusion of OH^–^, resulting in increased electrochemical reaction rates [44], [45]. Furthermore, the ultrasonic stirring effect induced by acoustic streaming can effectively suppress the accumulation and deposition of Al(OH)_3_ flocculates at the cathode interface, preventing electrode passivation and thus improving the operational stability and service lifetime of battery [26].

#### 3.2.2 EIS analysis

**Fig. 8:**
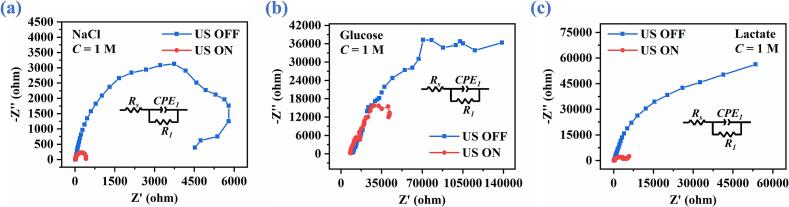
EIS of ABBB with and without ultrasound for NaCl, glucose, and lactate electrolytes, respectively.

The EIS tests are performed on the ABBB with and without ultrasonic excitation using NaCl, glucose, and lactate bioelectrolytes. The corresponding Nyquist plots are presented in Fig. 8(a)-Fig. 8(c). It is observed that the impedance arcs obtained under the ultrasound (US ON) are significantly reduced compared with those measured in the absence of ultrasound (US OFF), indicating a pronounced decrease in the overall interfacial impedance of battery system. Furthermore, the equivalent electrical circuits employed to fit the EIS spectra are shown in the insets of Fig. 8(a)-Fig. 8(c), where *R_s_* represents the internal resistance of battery, which mainly includes the bioelectrolyte resistance and contact resistance; *CPE_1_* denotes the constant phase element associated with the cathodic catalytic electrode; *R_1_* corresponds to the polarization resistance of cathode, incorporating both charge-transfer resistance and diffusion-layer resistance [46]. As such, the corresponding fitting parameters are summarized in Table 1.

**Table 1:** Fitting parameters of equivalent circuits for the measured EIS of ABBB.

Element		*R_s_* (Ω·cm^2^)	*R_1_* (Ω·cm^2^)	*CPE_1_* (Ω^−1^·S^n1^·cm^−2^)	*n_1_*
NaCl	US OFF	19	6755	1.72 × 10^-5^	0.853

US ON	12.8	607.2	2.03 × 10^-5^	0.826
Glucose	US OFF	9855	164,780	5.88 × 10^-6^	0.600
	US ON	7983	77,687	1.07 × 10^-5^	0.550
Lactate	US OFF	160.8	314,580	1.28 × 10^-5^	0.797
	US ON	126.7	6110	2.70 × 10^-5^	0.691

As shown in Table 1, for the NaCl bioelectrolyte, the solution resistance (*R_s_*) of battery decreases from 19 Ω·cm^2^ under US OFF to 12.8 Ω·cm^2^ under US ON, indicating that the ultrasonic stirring effect effectively reduces solution resistance by alleviating concentration polarization in the bioelectrolyte. Meanwhile, the polarization resistance (*R_1_*) of cathodic electrode is markedly reduced from 6755 Ω·cm^2^ to 607.2 Ω·cm^2^ upon the ultrasonic excitation. Similar trends are also observed for ABBBs using glucose and lactate bioelectrolytes. Specifically, for the glucose bioelectrolyte, *R_s_* and *R_1_* decrease from 9855 Ω·cm^2^ and 164780 Ω·cm^2^ to 7983 Ω·cm^2^ and 77687 Ω·cm^2^, respectively, when the ultrasonic liquid-phase catalysis is applied. For the lactate electrolyte, the solution resistance and polarization resistance are reduced from 160.8 Ω·cm^2^ and 314580 Ω·cm^2^ to 126.7 Ω·cm^2^ and 6110 Ω·cm^2^, respectively. These pronounced reductions in both solution resistance and polarization resistance demonstrate that the application of ultrasound is able to improve the catalytic performance of cathode electrode.

### 3.3 Effect of ultrasonic vibration velocity

**Fig. 9:**
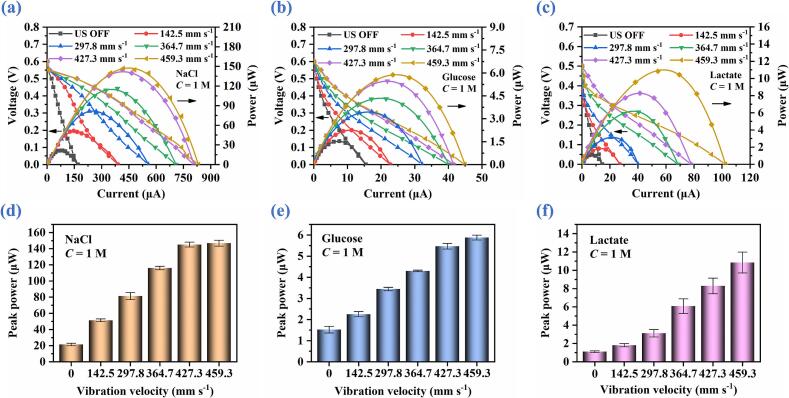
Discharge performance of ABBB under different ultrasonic vibration velocities in NaCl, glucose, and lactate bioelectrolytes. (a), (b) & (c) Polarization and power curves. (d), (e) & (c) Peak power versus vibration velocity.

Since the intensity of ultrasonic physical effects can be easily tuned by regulating the vibration velocity through adjustment of driving voltage applied to the ultrasonic transducer, the influence of ultrasonic vibration velocity on the performance of ABBB is systematically investigated. Polarization and power curves are measured at different vibration velocities (142.5, 297.8, 364.7, 427.3, and 459.3 mm/s) using 1-M NaCl, 1-M glucose, and 1-M lactate bioelectrolytes, and the corresponding results are shown in Fig. 9(a)-Fig. 9(c).

As summarized in Fig. 9(d)-Fig. 9(f), for NaCl-, glucose-, and lactate-based bioelectrolytes, the peak output power increases monotonically with the increasing vibration velocity. Specifically, the peak power rises from 51.37 mW (2.252 mW and 1.820 mW) under 142.5 mm/s to 146.754 mW (5.879 mW and 10.838 mW) under 459.3 mm/s for NaCl (glucose and lactate) bioelectrolytes.

Compared with the peak powers obtained under US OFF (21.383 mW, 1.522 mW, and 1.101 mW for NaCl, glucose, and lactate, respectively), the ultrasonic liquid-phase catalysis leads to progressively enhanced power outputs as the vibration velocity increases. At the vibration velocities of 297.8, 364.7, 427.3, and 459.3 mm/s, the peak powers are enhanced by 2.4- (1.5- and 1.7-fold), 3.8- (2.3- and 2.8-fold), 5.4- (2.9- and 5.5-fold), 6.7- (3.6- and 7.5-fold), and 6.9- (3.9- and 9.8-fold) for NaCl (glucose and lactate) electrolytes, respectively. This pronounced performance enhancement originates from the concurrent increase in acoustic pressure and acoustic streaming intensity at the catalytic electrode interface (Fig. S1).

### 3.4 Effect of electrode spacing

**Fig. 10:**
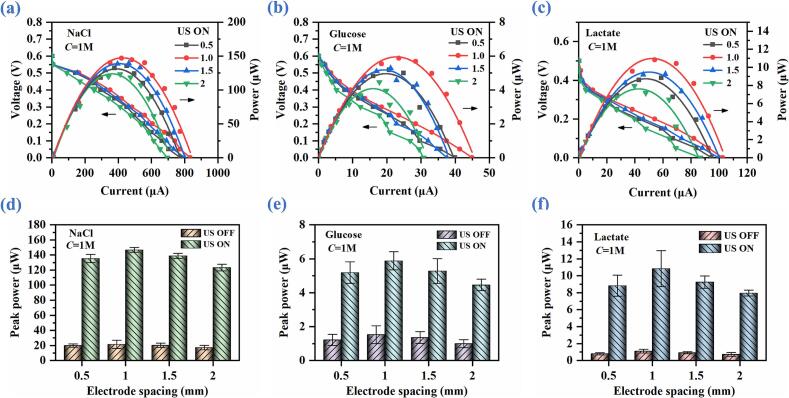
Discharge performance of ABBB without and with ultrasound catalysis under different electrode spacings of NaCl, glucose, and lactate bioelectrolytes. (a), (b) & (c) Polarization and power curves. (d), (e) & (c) Peak power versus electrode spacing.

To investigate the influence of electrode spacing on the discharge performance of ABBB, polarization and power curves are measured under the ultrasonic vibration velocity of 459.3 mm/s and the concentration of 1 M for different electrode spacings (0.5 mm, 1 mm, 1.5 mm, and 2 mm), and the results are presented in Fig. 10(a)-Fig. 10(c) and Fig. S2(a)-Fig. S2(c). Following which, the peak powers of ABBB versus electrode spacing are presented in Fig. 10(d)-Fig. 10(f). It is observed that the discharge performance of ABBB exhibits a non-monotonic variation trend with the change in electrode spacing for the three bioelectrolytes, showing the optimal performance under the electrode spacing of 1 mm.

The increase in peak power with the electrode spacing decreasing from 2 mm to 1 mm can be attributed to the shortened ion transport paths between the anode and cathode [29]. That is, a smaller electrode spacing can effectively reduce the ohmic resistance of bioelectrolyte in the inter-electrode region, thereby accelerating the migration of ions between the two electrodes [47].

However, when the electrode spacing is further decreased to 0.5 mm, the discharge performance of ABBB decreases as well. This is due to that the ultra-small electrode spacing can easily cause the accumulation of Al hydroxide flocculates in the confined inter-electrode region, which further aggravates the concentration polarization at the electrode interface [48].

### 3.5 Effect of bioelectrolyte concentration

**Fig. 11:**
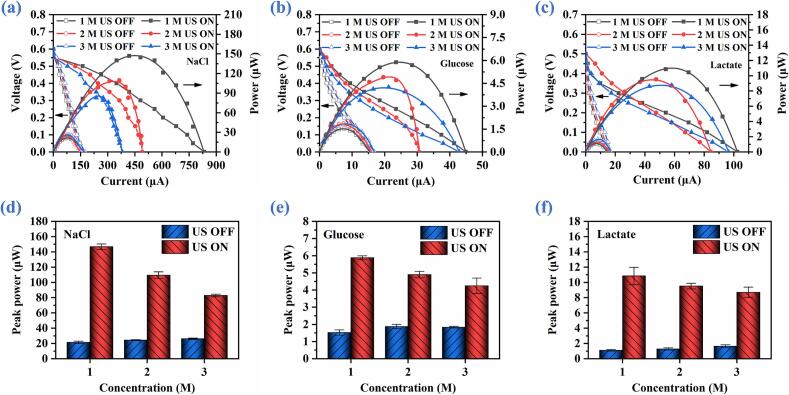
Discharge performance of ABBB without and with ultrasound catalysis under different concentrations of NaCl, glucose, and lactate bioelectrolytes. (a), (b) & (c) Polarization and power curves. (d), (e) & (c) Peak power versus concentration.

Discharge performance of ABBB under different bioelectrolyte concentrations is further investigated using NaCl, glucose, and lactate bioelectrolytes in conjunction with the ultrasound of 65.12 kHz and 459.3 mm/s. Fig. 11(a)-Fig. 11(c) present the polarization curves of ABBB with and without ultrasound under the bioelectrolyte concentrations of 1 M, 2 M, and 3 M, respectively. Under US OFF, when the bioelectrolyte concentration is increased from 1 M to 3 M, the peak power rises from 21.383 mW to 26.137 mW for NaCl, from 1.522 mW to 1.836 mW for glucose, and from 1.101 mW to 1.631 mW for lactate, meaning that the discharge performance of ABBBs is improved by 22.2% (NaCl), 20.6% (glucose), and 48.1% (lactate), respectively. These improvements can be attributed to the increased ionic conductivity of bioelectrolyte at higher concentrations, which can reduce the internal resistance (*R_s_*) and enhance mass transport, thereby accelerating electrochemical reaction kinetics at the THRI [30], [49].

Peak powers of ABBB under US OFF and US ON are summarized in Fig. 11(d)-Fig. 11(f). Compared with the US OFF counterparts, the ultrasonic excitation leads to substantial power enhancement across all concentrations. Specifically, the peak power is increased by 6.7- (3.9- and 9.4-fold), 4.5- (2.6- and 7.4-fold), and 3.2- (2.3- and 5.3-fold) under the bioelectrolyte concentrations of 1 M, 2 M, and 3 M for NaCl (glucose and lactate), respectively.

Notably, although the ultrasonic catalysis enhances battery performance at all tested concentrations, an inverse trend is observed with the increasing bioelectrolyte concentration, that is, the peak power gradually decreases as the bioelectrolyte concentration increases. For example, the peak power decreases from 146.754 mW to 82.868 mW for NaCl, from 5.879 mW to 4.248 mW for glucose, and from 10.838 mW to 8.699 mW for lactate, as the concentration increases from 1 M to 3 M. We attribute this phenomenon primarily to the increased viscosity of bioelectrolytes at higher concentrations (Table 2). Moreover, these increasing bioelectrolyte viscosities lead to a significant reduction in acoustic streaming velocity (Fig. S3), thereby weakening the catalytic effect enabled by the acoustic streaming component.

**Table 2:** Relationship between the viscosity and concentration of bioelectrolyte.

Type	1 M	2 M	3 M
NaCl	1.25 mPa·S	1.43 mPa·S	1.52 mPa·S
Glucose	1.63 mPa·S	3.21 mPa·S	7.75 mPa·S
Lactate	1.31 mPa·S	1.65 mPa·S	1.78 mPa·S

### 3.6 Effect of bioelectrolyte type

Building upon the above investigations into the regulation of ABBB performance through the ultrasonic vibration velocity and electrolyte concentration, this section further examines the influence of different bioelectrolyte types on battery discharge behaviors under the ultrasound of 65.12 kHz and 459.3 mm/s. The selection of bioelectrolyte is motivated by the typical compositions of human sweat, which primarily includes urea, glucose, ascorbic acid (AA), uric acid (UA), lactate, KCl, and NaCl with the approximate concentrations of 30 mM, 50 μM, 20 μM, 4 mM, 5 mM, 10 mM, and 100 mM, respectively, as well as artificial sweat and natural human sweat samples (Vol-1 and Vol-2). Utilizing these sweat-derived biocomponents as electrolytes therefore represents a promising strategy for developing self-powered biosensing systems.

**Fig. 12:**
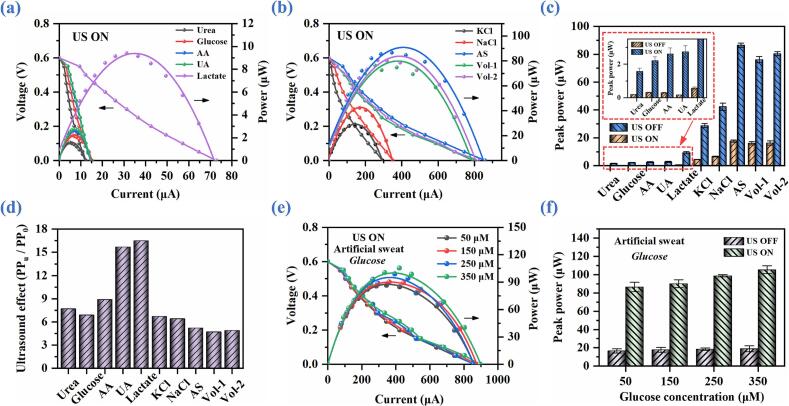
Discharge performance of ABBB with ultrasonic catalysis for different bioelectrolyte types. (a) & (b) Polarization and power curves. (c)-(d) Peak power and ultrasound-induced performance enhancement versus bioelectrolyte type. (e)-(f) Polarization curves and peak power versus different concentrations of glucose in artificial sweat.

Given that battery discharge performance is strongly dependent on electrolyte composition, systematic discharge tests are conducted using these biolectrolytes, artificially synthesized sweat with concentrations matching those of human sweat, and natural human sweat. The corresponding *I*-*V* and *I*-*P* characteristics of ABBB are shown in Fig. 12(a) and Fig. 12(b), respectively. Under US OFF, the peak powers of ABBB using AA, UA, artificial sweat, Vol-1, and Vol-2 as bioelectrolytes are approximately 0.295 μW, 0.319 μW, 16.611 μW, 16.098 μW, and 16.401 μW, respectively (Fig. S4(a), Fig. S4(b), and Fig. 12(c)). Under US ON, the corresponding peak powers are significantly enhanced to 2.616 μW, 2.748 μW, 81.520 μW, 76.227 μW, and 80.028 μW, showing that the peak powers of ABBB are increased by approximately 8.87-, 8.61-, 4.91-, 4.74-, and 4.89-fold, respectively. The slight decrease in peak power of natural human sweat compared with the artificial sweat is mainly attributed to the compositional variability and individual difference in human sweat samples. To characterize the influence of compositional variability, we perform tests using artificial sweat with gradient glucose concentrations (50 μM, 150 μM, 250 μM, 350 μM), and the results are presented in Fig. 12(e) and Fig. 12(f). These results indicate that the variation of glucose concentration in artificial sweat can induce shifts in polarization curves, and the peak power slightly increases with the increase in concentration.

Additionally, it is observed that the performance enhancement of ABBB peak power enabled by ultrasound can be even higher than 15 times for UA and lactate, as observed from Fig. 12(d). These results convincingly demonstrate that the bioelectrolytes derived from sweat can effectively support and significantly enhance the discharge performance of ABBBs through our ultrasonic liquid-phase catalysis strategy. The integration of ultrasonic liquid-phase catalysis with sweat bioelectrolytes not only overcomes the intrinsic performance limitations of conventional ABBBs but also is promising for establishing a new class of self-powered biosensing systems.

## 4 Conclusion

Here, we proposed and experimentally demonstrated an ultrasonic liquid-phase catalysis strategy to significantly enhance the discharge performance of ABBB. By integrating ultrasonic excitation with bioelectrolytes derived from human sweat components, we establish a general and effective approach to overcoming the intrinsically low discharge performance of conventional metal-based bioelectrolyte batteries. FEM simulations and EIS analyses reveal that the performance enhancement originates from the synergistic effects of acoustic pressure, acoustic streaming, and ultrasonic stirring. Importantly, among the bioelectrolytes mimicking human sweat components, artificial sweat exhibits stable and favorable discharge performance under ultrasonic liquid-phase catalysis, with the peak output power enhanced by around 5 folds. These results convincingly demonstrate that sweat-derived bioelectrolytes can serve as viable and efficient electrolytes for ABBs with the assistance of ultrasound.

Beyond performance enhancement, this work establishes a new paradigm for self-powered biosensing, in which biological fluids act simultaneously as both the sensing target and the energy source, with ultrasonic excitation serving as an effective performance-enhancing mechanism, providing a solid basis for the development of bio-friendly and self-powered systems for wearable and portable electronics.

## CRediT authorship contribution statement

**Huiyu Huang:** Writing – original draft, Project administration, Funding acquisition, Formal analysis, Data curation, Conceptualization. **Jia Yin:** Writing – review & editing. **Shuo Zhang:** Writing – review & editing. **Quanquan Yang:** Writing – review & editing, Funding acquisition. **Zhong Chen:** Writing – review & editing, Funding acquisition. **Qiang Tang:** Writing – review & editing, Funding acquisition. **Xiaomin Qi:** Writing – review & editing. **Songfei Su:** Writing – review & editing. **Jinyan Chen:** Writing – review & editing, Funding acquisition. **Hao Chen:** Writing – review & editing. **Kan Zhu:** Writing – review & editing. **Shengling Qu:** Writing – review & editing. **Pengzhan Liu:** Writing – review & editing, Supervision, Formal analysis.

## Declaration of competing interest

The authors declare that they have no known competing financial interests or personal relationships that could have appeared to influence the work reported in this paper.
