# Supplementary material for: Ultrasonic liquid-phase catalysis for enhanced power generation in Al-based bioelectrolyte batteries
Source: Ultrason Sonochem. 2026 Mar 15;128:107822. doi: 10.1016/j.ultsonch.2026.107822 (PMC13011236; doi:10.1016/j.ultsonch.2026.107822)
Supplement: Supplementary Data 1 [file mmc1.docx]

**Supplementary material**

**Ultrasonic liquid-phase catalysis for enhanced power generation in Al-based bioelectrolyte batteries**

Huiyu Huang^1*^, Jia Yin^2^, Shuo Zhang^2^, Quanquan Yang^1^, Zhong Chen^1^, Qiang Tang^1^, Xiaomin Qi^3^, Songfei Su^4^, Jinyan Chen^5^, Hao Chen^1^, Kan Zhu^1^, Shengling Qu^1^, Pengzhan Liu^6,7*^

1 Jiangsu Key Laboratory of Advanced Manufacturing Technology, Faculty of Mechanical and Material Engineering, Huaiyin Institute of Technology, Huaian, 223003, China

2 State Key Laboratory of Mechanics and Control for Aerospace Structures,

Nanjing University of Aeronautics and Astronautics, Nanjing 210016, China

3 School of Mechanical and Automotive Engineering, Anhui Polytechnic University, Wuhu 241000, China

4 School of Mechanical Engineering, Nanjing Institute of Technology, Nanjing 211167, China

5 School of Automotive Engineering, Changzhou Institute of Technology, Changzhou 213032, China

6 School of Mechanical and Aerospace Engineering, Nanyang Technological University, 639798, Singapore

7 School of Materials Science and Intelligent Engineering, Nanjing University, Suzhou 215163, China

*Corresponding author’s email address: hyhuang@nuaa.edu.cn; PENGZHAN001@e.ntu.edu.sg

**Table S1****. Performance parameters of the ultrasonic transducer.**

| Symbol | Parameter | Value |
| --- | --- | --- |
| *f* | Resonance frequency | 65 ± 1.5 kHz |
| *C* | Electrostatic capacitance | 2300 ± 10 % pF |
| *F* | Resonance impedance | ≤ 35 Ω |
| *SIR* | Insulation resistance | ≥ 100 MΩ |
| *D × H* | Diameter and height | 25 *×* 31 mm |

**Table S2. Material parameters of bioelectrolytes used in the simulations.**

| Symbol | Quantity | Value for bioelectrolyte | | | Value for electrode | | Value for glass substrate |
| --- | --- | --- | --- | --- | --- | --- | --- |
|  |  | NaCl | Glucose | lactate | Al | Pt |  |
| *ρ* | Density (kg/m^3^) | 1550 | 1580 | 1209 | 2700 | 21389 | 2203 |
| *μ* | Dynamic viscosity (Pa∙s) | 1.25 | 1.63 | 1.31 | / | / | / |
| *c* | Sound speed (m/s) | 1500 | 1500 | 1500 | / | / | / |
| *E* | Young’s modulus (GPa) | / | / | / | 69.1 | 158.1 | 73.1 |
| *μ_p_* | Poisson's ratio | / | / | / | 0.331 | 0.346 | 0.17 |





Fig. S1. Acoustic pressure amplitude and acoustic streaming versus ultrasonic vibration velocity.


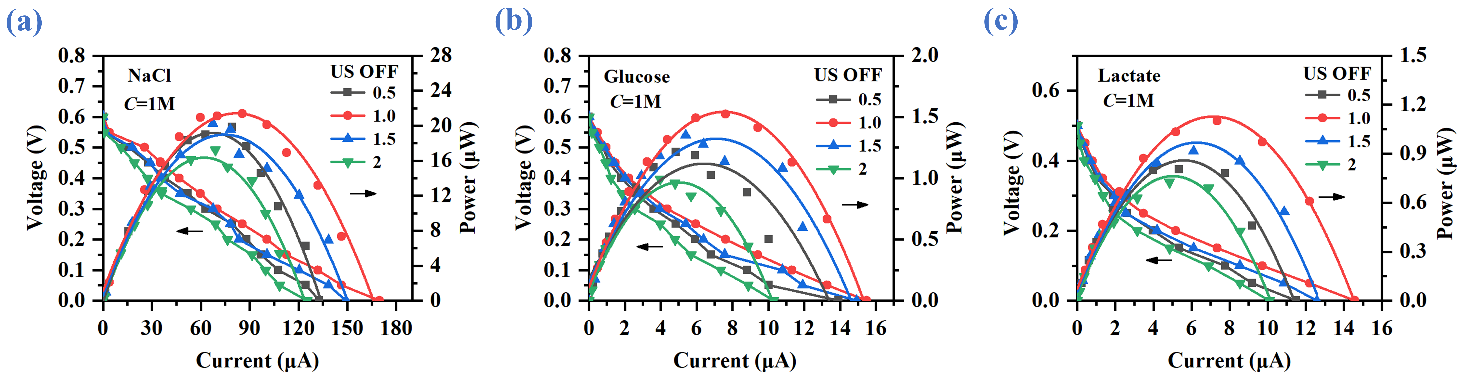


Fig. S2. Discharge performance of ABBB without ultrasound under different electrode spacings. (a), (b) & (c) Polarization and power curves for NaCl, Glucose, and Lactate.





Fig. S3. Acoustic streaming velocity versus bioelectrolyte concentration.


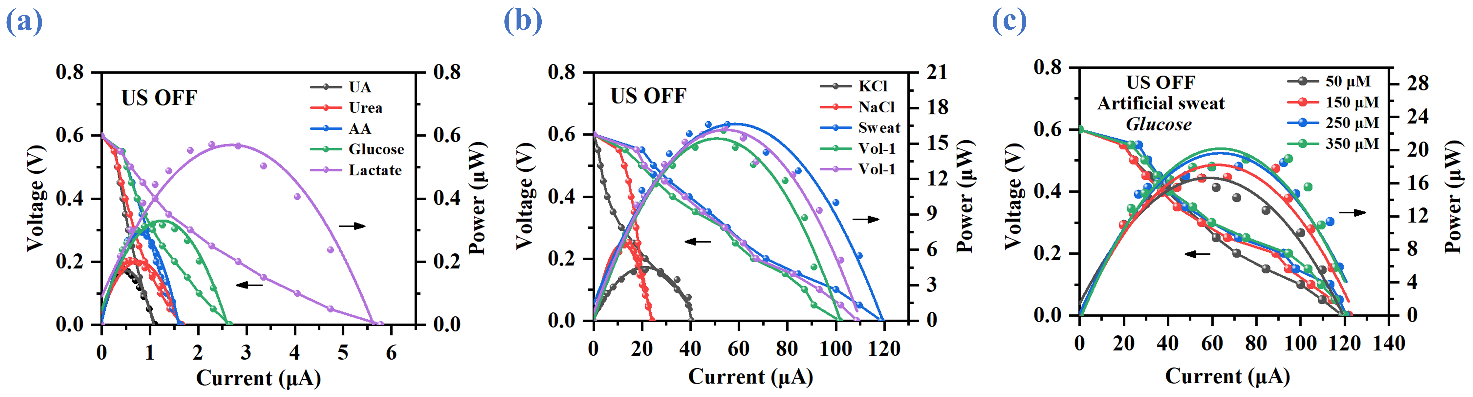


Fig. S4. Discharge performance of ABBB without ultrasound for different bioelectrolyte types. (a) & (b) Polarization and power curves. (c) Polarization curves under different concentrations of glucose in artificial sweat.
